# Association of Age with Outcomes in Adrenocortical Carcinoma: A Combined Cancer Registry and Multi-Omic Analysis

**DOI:** 10.3390/cancers18091483

**Published:** 2026-05-05

**Authors:** Lindsay F. Remer, Rachyl M. Shanker, Thomas J. Meyer, John I. Lew, Naris Nilubol

**Affiliations:** 1 Surgical Oncology Program, National Cancer Institute, National Institutes of Health, Bethesda, MD 20892, USA; 2 Advanced Biomedical Computational Science, Frederick National Laboratory for Cancer Research, Frederick, MD 21702, USA; 3 CCR Collaborative Bioinformatics Resource, Center for Cancer Research, National Cancer Institute, National Institutes of Health, Bethesda, MD 20892, USA; 4 Division of Endocrine Surgery, DeWitt Daughtry Family Department of Surgery, University of Miami Leonard M. Miller School of Medicine, Miami, FL 33125, USA

**Keywords:** adrenocortical carcinoma, menopause, age, overall survival

## Abstract

**Background:** Given the higher incidence of adrenocortical carcinoma in peri-menopausal women, this study investigates the associations between sex, age, and ACC prognosis. **Methods:** We analyzed the National Cancer Database (NCDB) for adult patients with ACC (*n* = 2384) from 2004 to 2022. Overall survival (OS) was the primary endpoint. Patients were stratified by sex and menopausal status (pre- and post-menopausal age of ≤45 and ≥55, respectively). Data from The Cancer Genome Atlas (TCGA) cohort (*n* = 92) were analyzed to examine clinical characteristics, genetics, transcriptomics, and methylation profiles of primary ACC samples by age group. **Results:** The analysis of the NCDB cohort revealed that younger men and pre-menopausal women had significantly longer OS compared to older groups. However, age was the only independent variable associated with OS. Using 50 years as the optimal age cutoff, we found no differences in clinical characteristics, rates of pathogenic driver mutations, methylation or gene expression profiles, or enriched molecular pathways in the TCGA ACC cohort by age group. **Conclusions:** While age ≥50 years is an independent factor associated with shorter OS in ACC, no differences in clinical characteristics or multi-omic profiles were observed by age. These findings suggest that age-related prognosis may be influenced by other factors such as microenvironmental changes or epigenetics.

## Simple Summary

Adrenocortical carcinoma (ACC) is a rare malignancy more common among women, with a peak incidence at 40–50 years of age. We hypothesized that there was an association between female sex hormones and the incidence of ACC. The National Cancer Database was queried for ACC patients, and we found that age is the only independent variable associated with overall survival. A receiver operating characteristic (ROC) curve with Youden’s index generated an optimal age cutoff, separating patients by age ≥50 years. ACC patients in The Cancer Genome Atlas were examined, though no differences in clinical characteristics, rates of pathogenic driver mutations, differential methylation profiles, or differentially expressed genes existed between age groups. While age ≥50 years is an independent factor associated with shorter overall survival in ACC, no differences in clinical characteristics, tumor genetics, gene expression, or methylation profiles were observed by age. These findings suggest that age-related prognosis may be influenced by other factors.

## 1. Introduction

Adrenocortical carcinoma (ACC) is a rare and aggressive malignancy, generally with a poor prognosis because of the limited efficacy of current treatments. Incidence is estimated to be about 0.7 to two cases per 100,000 person-years and has two peak incidences: one in the first decade of life and the other between 40 and 50 years [1,2]. The disease is also more common among women [2]. Five-year survival rates range from 16% to 47%, depending on the tumor stage at presentation [3]. Complete surgical resection offers the best chance for long-term survival but is reserved for those with localized disease [4,5,6].

Previous studies using databases and single-institution series have found increasing age, higher Charlson–Deyo Comorbidity Index (CDCI) scores, high tumor grade, lack of surgical resection, and positive margins after resection to be associated with shorter survival [1,7]. Sex has not proven to be associated with outcomes despite the female predominance. While most tumors show a male predominance, all adrenal tumors, regardless of histologic type, are more frequent in women [5,8,9]. ACC has a predilection for females with a female-to-male ratio documented to be 1.5–2.5:1 [10,11]. The role of estrogens in ACC remains to be seen, with some noting a relative increase in ACC diagnosis in pregnancy [12]. Moreover, in vitro and in vivo experiments using the ACC cell line NCI-H295R revealed that proliferation was inhibited with the use of the estrogen-related receptor alpha (ERRα) inverse agonist XCT790 [13]. Estrogens have also been shown to play a role in mesothelioma, meningioma, prostate cancer, renal cell carcinoma, colorectal and lung cancer [14]. Thus, given the increased incidence of ACC among women and peak diagnosis around peri-menopause, this study investigates the potential associations between overall survival (OS) and sex, including menopausal status and age, in ACC.

## 2. Methods

### 2.1. Data Source and Study Population

A retrospective analysis using data from the NCDB for the years 2004 to 2022 was conducted. The NCDB is a joint venture between the Commission on Cancer for the American College of Surgeons and the American Cancer Society. The data collection, integrity of data, and quality have been previously documented [15]. Because data from the NCDB is deidentified, the institutional review board of the primary author’s institution exempted the study from review.

Between the years of 2004 and 2022, adult patients (≥18 years of age) with ACC were identified using the International Classification of Diseases for Oncology, Third Edition, code 8370/3 and site-specific code of C74.

We accessed the gene expression data of primary ACC samples from the Firehose Legacy ACC cohort (*n* = 92) in The Cancer Genome Atlas (TCGA) database via the cBioportal website (https://www.cbioportal.org/study/summary?id=acc_tcga. accessed on 17 April 2025). Within this cohort, pathogenic driver mutations (*n* = 90), transcriptomics (*n* = 79), and methylation profiles (*n* = 79) were analyzed.

### 2.2. Covariates

Covariates used in the analysis of NCDB data included patients’ demographics (age, sex, race, Charlson–Deyo Comorbidity Index (CDCI) score), year of diagnosis, tumor characteristics, and type of therapy received.

Covariates used in the analysis of TCGA data included patients’ age, tumor subtype, as previously categorized by de Reyniés et al. and imported and aligned using sample identifiers from Zheng et al. (C1A-aggressive, C1B-indolent), methylation status, driver mutation, tumor stage, metastasis, grade, treatment with mitotane, and tumor functional status [16,17].

### 2.3. Statistical Analysis

SPSS version 30.0 (IBM, Armonk, NY, USA) was used for statistical analyses. The independent samples *t*-test was used to compare continuous variables. The chi-square test was used to compare categorical data. Female patients were initially split into two categories: pre-menopausal (≤45 years old) and post-menopausal (≥55 years old). Peri-menopausal (between 45 and 55 years old) women were excluded from the initial analysis. The overall survival (OS) by age group and sex was estimated and compared using the Kaplan–Meier method with the Log-rank test. The Cox proportional-multivariable hazards regression model was used to determine which independent variables were associated with overall survival. Age was modeled as a continuous variable. Non-linearity was assessed by including a quadratic term (age^2^) in the Cox model. Missing data were handled using complete-case analysis (listwise deletion), whereby only observations with non-missing values for all covariates were included in the multivariable Cox models. A receiver operating characteristic (ROC) curve with Youden’s index was generated to identify the optimal age cutoff for overall survival that was later used to dichotomize the NCDB and TCGA cohorts. Multiple imputation was considered, but given the high proportion of missing data for stage and grade in the NCDB, imputation was deemed unlikely to be reliable and was not performed.

ACC TCGA bulk RNA sequencing data analysis was performed with the NIH Integrated Data Analysis Platform (NIDAP, Bethesda, MD, USA) using R programs (IBM Corp., Armonk, NY, USA) developed on the Foundry platform (Palantir Technologies, Denver, CO, USA). NIDAP was used for downstream gene set enrichment analysis (GSEA). The gene counts matrix was imported into the NIDAP platform, where genes were aligned to the human reference genome (GRCh38), filtered for low counts (<1 Cpm), and normalized by quantile normalization using the limma package [18]. Using limma-Voom, differentially expressed genes were calculated. Genes with an adjusted *p*-value < 0.05 and an absolute log2 fold change greater than 1 were considered significantly differentially expressed. GSEA was performed using the fgsea package [19] and the Broad Institute Molecular Signature Database (mSigDB) “Hallmark Pathways” gene set to cover a wide array of cellular processes. Pathways with adjusted *p*-values < 0.05 were considered statistically significantly de/activated.

Two-tailed *p*-values below 0.05 were considered statistically significant.

## 3. Results

### 3.1. Baseline Characteristics

A total of 2834 patients with ACC between 2004 and 2022 from the NCDB cohort met the inclusion criteria. Overall, 60.7% of the included patients were female. The majority of patients were white (84%), followed by black (10%), Asian and Pacific Islander (3%). The median age at diagnosis was 56, ranging between 18 and 90 years old. The baseline characteristics of the entire cohort are summarized in Table 1.

### 3.2. Characteristics of Study Cohorts

**Figure 1:**
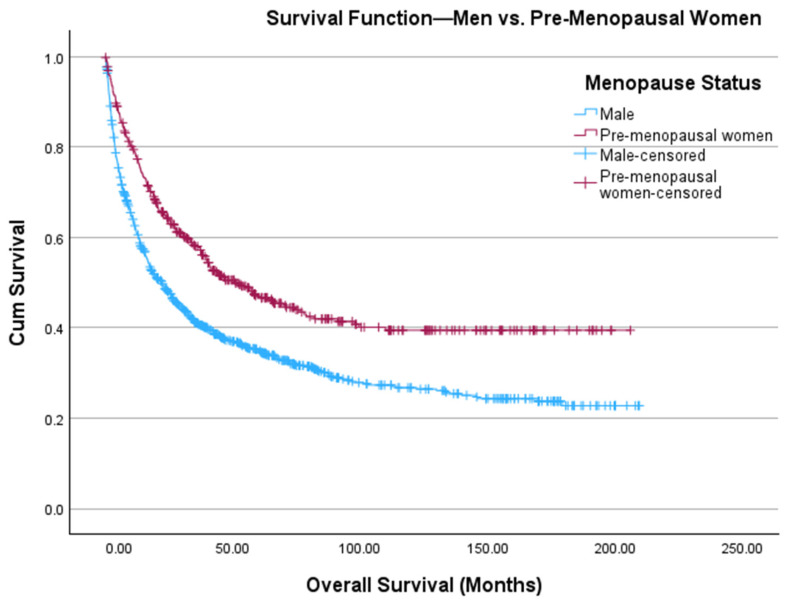
Kaplan–Meier curve depicting OS of men (blue, *n* = 1115) and pre-menopausal women (red, *n* = 486) diagnosed with ACC. The *x*-axis depicts time in months, and the *y*-axis shows cumulative survival probability. Tick marks denote censored observations. Survival distributions between groups are visually compared across the follow-up period.

**Figure 2:**
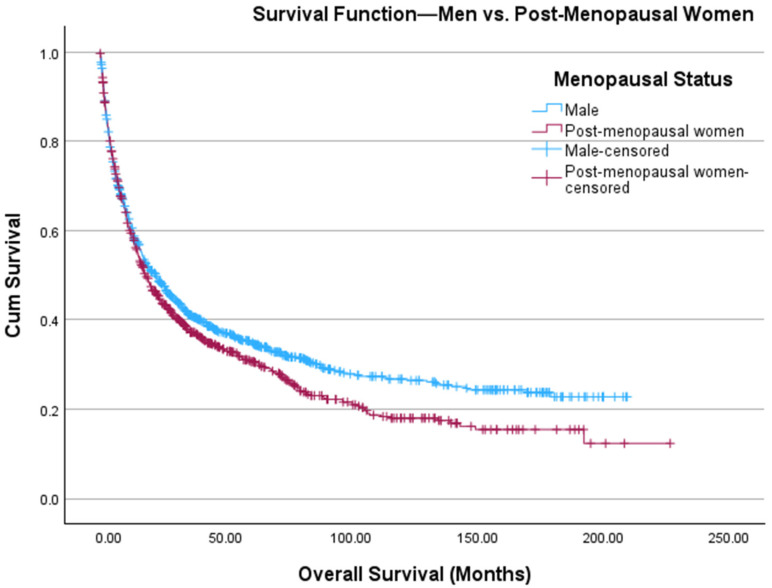
Kaplan–Meier curve depicting OS of men (blue, *n* = 1115) and post-menopausal women (red, *n* = 937) diagnosed with ACC. The *x*-axis depicts time in months, and the *y*-axis shows cumulative survival probability. Tick marks denote censored observations. Survival distributions between groups are visually compared across the follow-up period.

**Figure 3:**
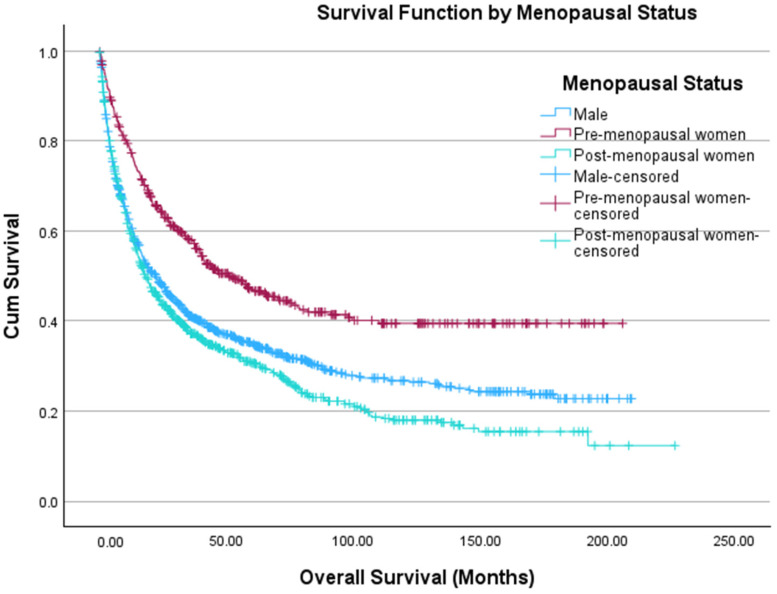
Kaplan–Meier curve depicting OS of men (blue, *n* = 1115) and pre- (red, *n* = 486) and post-menopausal women (green, *n* = 937) diagnosed with ACC. The *x*-axis depicts time in months, and the *y*-axis shows cumulative survival probability. Tick marks denote censored observations. Survival distributions between groups are visually compared across the follow-up period.

There were 486 and 937 women who were considered pre-menopausal (age ≤ 45 years) and post-menopausal (age ≥ 55 years), respectively. Table 2 offers a comparison between men, pre-menopausal women, and post-menopausal women. Pre-menopausal women had significantly higher rates of being Hispanic (*p* = 0.02) and receiving surgical (*p* < 0.001) and systemic treatments (*p* < 0.001), but had significantly lower CDCI scores (*p* < 0.001) than men. Men had significantly higher rates of being Hispanic (*p* = 0.004) and receiving systemic therapy (*p* < 0.001) than post-menopausal women. We found that pre-menopausal women had higher estimated OS than men (97.7 months vs. 69.4 months, *p* < 0.001) (Figure 1). However, men were found to have higher estimated OS than post-menopausal women (69.4 months vs. 58.1 months, *p* = 0.021) (Figure 2). The Kaplan–Meier curves in Figure 1 and Figure 2 were combined in Figure 3. On multivariate Cox regression, menopausal status was the only statistically significant independent variable associated with overall survival (Table 3). In a sensitivity analysis restricted to patients with known stage and CDCI score, the results were consistent with the primary analysis shown in Table 3 (Supplemental Table S2 and Supplemental Table S3, respectively).

**Figure 4:**
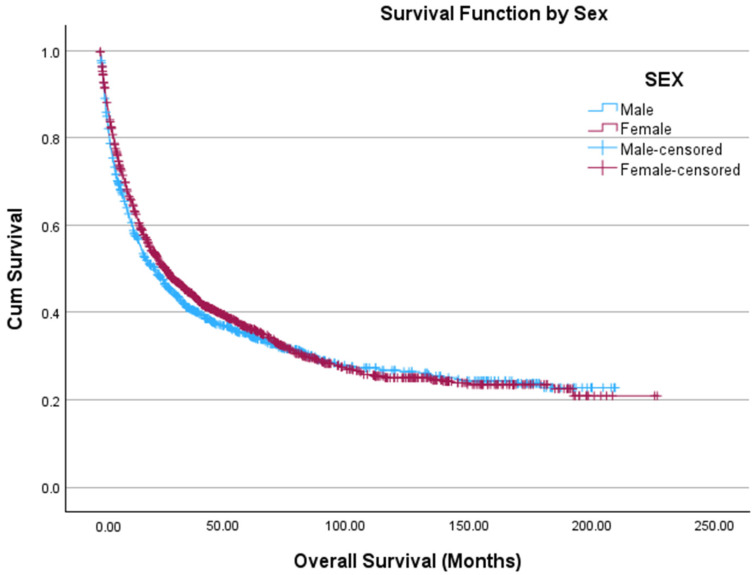
Kaplan–Meier curve depicting OS of all men (blue, *n* = 1115) and women (red, *n* = 1269) diagnosed with ACC. The *x*-axis depicts time in months, and the *y*-axis shows cumulative survival probability. Tick marks denote censored observations. Survival distributions between groups are visually compared across the follow-up period.

To ensure that the outcome of this analysis was not simply related to age or sex differences, a Kaplan–Meier curve was generated for all men and women, without excluding any participants based on age, in the cohort with no significant difference noted between men and women (69.4 months vs. 74.0 months, *p* = 0.167) (Figure 4). Then, men were categorized into the same age groups as women by menopausal status. Pre-menopausal women had higher estimated OS than post-menopausal women (97.7 months vs. 58.1 months, *p* < 0.001), young men (97.7 months vs. 84.0 months, *p* = 0.008), and older men (97.7 months vs. 57.8 months, *p* < 0.001). Young men had higher estimated OS than post-menopausal women (84.0 months vs. 58.1 months, *p* < 0.001) and older men (84.0 months vs. 57.8 months, *p* < 0.001). We found no differences in OS between men and women ≥55 years old. (Supplemental Figure S1).

**Figure 5:**
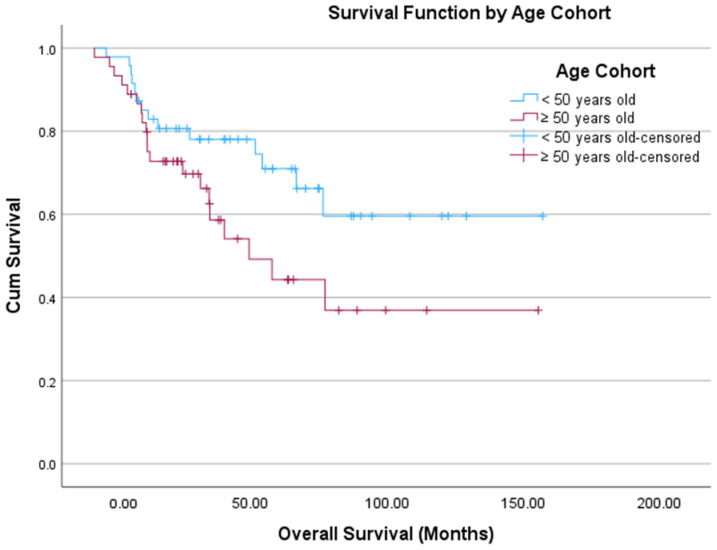
Kaplan–Meier curve depicting OS by age cohort: young (<50 years old, blue) and old (≥50 years old, red) diagnosed with ACC. The *x*-axis depicts time in months, and the *y*-axis shows cumulative survival probability. Tick marks denote censored observations. Survival distributions between groups are visually compared across the follow-up period.

Next, a receiver operating characteristic (ROC) curve with Youden’s index was performed, which identified an age of 50 years as an optimal cutoff associated with OS. Table 4 offers a comparison between patients <50 years old and those ≥50 years old. There were significantly more patients in the younger cohort who were Hispanic (*p* < 0.001), had lower CDCI scores (*p* < 0.001), and who received surgery (*p* < 0.001) and systemic therapy (*p* < 0.001). Figure 5 displays the Kaplan–Meier curve for these two groups, though there was no statistically significant difference in the estimated OS between the young and old groups (107.5 months vs. 78.2 months, *p* = 0.07). On multivariate Cox regression, age (*p* < 0.001), CDCI score (*p* = 0.028), and surgery (*p* < 0.001) were statistically significant predictors of outcome (Table 5). We also performed a sensitivity analysis restricted to patients with a CDCI score of 0 (no comorbidities) (Supplemental Table S4). The results were consistent with the primary analysis, with age and surgery being statistically significant predictors of outcome, supporting the robustness of our findings. The quadratic term for age was not statistically significant, supporting a linear relationship. In a sensitivity analysis restricted to patients with known stage, the results were consistent with the primary analysis shown in Table 5 (Supplemental Table S5).

To assess whether the difference in OS by age group was due to different ACC biology, we compared clinical features and molecular characteristics of ACC in TCGA cohort by age group, using 50 years as a cutoff. We found no statistically significant differences in clinical features and outcomes (Table 6). In addition, the rates of known and recurrent driver mutations in ACC by age group were not statistically significantly different (Supplemental Table S1).

### 3.3. GSEA and Pathway Analysis

Finally, we analyzed the TCGA ACC dataset to identify differentially expressed genes between age groups. No individual genes met our predefined thresholds for statistical significance (adjusted *p*-value < 0.05 and absolute log2 fold change >1). Nevertheless, because pathway-level changes can emerge even when single-gene differences do not reach significance, we used the full ranked gene expression profile, based on unadjusted differential expression *p*-values, as input for pathway enrichment analysis. This allowed us to evaluate whether coordinated changes in gene expression were present between individuals <50 years old and those ≥50 years old by testing differentially enriched Hallmark Pathways, despite the absence of strongly differentially expressed individual genes. Supplemental Figure S2 displays the six pathways found to be differentially expressed with an adjusted *p*-value < 0.05, though there is an absence of pathways associated with aggressive cancer behavior, such as oncogenic signaling, cell growth, cell invasion and immune suppression.

## 4. Discussion

ACC is a devastating disease with limited treatment options and poor five-year survival. Adrenal tumors are more common among women, with ACC being no exception. Some studies have suggested that sex hormones may play a role in tumorigenesis and tumor progression in ACC [12,13]. This study retrospectively reviews NCDB data from 2004 to 2022 and TCGA ACC data, and performs GSEA and pathway analysis to better understand prognostic indicators in patients with ACC. We hypothesized that estrogens and, as a surrogate, menopausal status may influence survival, as ACC is more prevalent among women and exhibits a peak within the peri-menopausal age range [1,2,10,11]. However, analysis of NCDB data revealed that differences in survival were a function of age. The age cutoff generated from NCDB data (age < or ≥50 years old) is consistent with the one used by the European Network for the Study of Adrenal Tumors (ENSAT) for the S-GRAS point-based scoring system evaluated for prognostic stratification of ACC patients [20].

The CDCI score and whether patients underwent surgery were also predictive of survival; however, as we know, the more comorbidities, the less likely a patient is to be offered surgery, and they may experience more challenges in their diagnosis and care [21]. Additionally, greater than 90% of older adults with cancer also have one or more chronic comorbidities, which impact treatment exposure, quality of life, and ultimately survival [22,23]. Of note, in a sensitivity analysis for the Cox regression shown in Table 5, when restricted to only patients with known stage and tumor grade, the CDCI score was no longer a significant predictor of survival and may have been confounded by these variables. In this study, we identified that age is a prognostic indicator of survival in ACC. Our bioinformatic analysis showed that ACC transcriptomics do not differ by age group, suggesting that age, not molecular features of ACC, played an important role in overall survival. This discovery warrants further investigation.

There were no statistically significant differences noted between age groups when TCGA ACC data were analyzed. This may be a result of a small sample size, resulting in a type II statistical error, and the lack of transcriptomic differences does not exclude microenvironmental or epigenetic effects.

Age-related differences in survival may be mediated through immunosenescence and concurrent remodeling of the tumor microenvironment. Aging is associated with the accumulation of oncogenic mutations and the development of a more permissive environment for tumorigenesis [24]. At the immune level, aging alters T cell development and function, characterized by reduced naïve T cell populations, expansion of exhausted and memory T cells, and impaired cytotoxic activity [25]. In parallel, structural changes in the extracellular matrix (ECM), including increased rigidity and loss of integrity, may facilitate tumor progression and contribute to epithelial-to-mesenchymal transition (EMT) [26]. Cancer-associated fibroblasts (CAFs) further remodel the ECM, and in the aged tumor microenvironment, senescent CAFs exhibit an altered secretome that promotes tumor growth and may influence immune checkpoint efficacy [26]. Within the adrenal cortex, which is relatively immunosuppressed due to the presence of glucocorticoids, myeloid cells, like macrophages, remain abundant; however, their phagocytic function declines with age, further impairing effective anti-tumor immune responses [27].

Although the incidence of ACC is higher among women, the mechanisms underlying this sexual dimorphism remain incompletely understood, as demonstrated in this study. The adrenal gland is one of the most sexually dimorphic non-reproductive organs [28]. Androgen receptor (AR) signaling has been shown to directly suppress proliferation in the adrenal cortex in a mouse model [28]. Furthermore, male ACC patients have a more prominent phagocytic macrophage signature than female ACC patients, suggesting sex-specific differences in immune regulation within the adrenal microenvironment [11]. Mouse models further support a role for myeloid immune responses in adrenal tumorigenesis, partially regulated by male sex steroid hormones [27,29]. While these findings indicate that sex hormones may influence tumor initiation or early progression, as we hypothesized, the age-associated immune and microenvironmental changes described above may play a more dominant role in shaping outcomes. Future investigations are needed to delineate the interaction between aging, immune function and sex hormone signaling in ACC.

Currently, differentiated thyroid cancer (DTC) is the only malignancy that includes age as a component of the American Joint Committee on Cancer (AJCC) staging system, with an age cutoff of 55 years in the most recent edition [30]. In these malignancies, age has been found to be an independent predictor of survival. The underlying mechanisms for this phenomenon have yet to be identified, with work identifying that age is associated with variable expression of the sodium–iodine symporter and the presence of BRAF V600E mutations, leading to increased risk of cancer-related mortality that increases with age [31,32,33]. Ebbehoj et al. explored population records in Olmstead County, MN, from 1 January 1995 to 31 December 2017 and found that more than 90% of adrenal tumors are diagnosed in patients older than 40 years [34]. Within the adrenal cortex, tumorigenesis may be driven by both genetic mutations and remodeling of the tissue microenvironment [27]. As cells become senescent, they activate a hypersecretory state that influences the tissue microenvironment [29,35]. However, in the adrenals, glucocorticoids may suppress this hypersecretory state [36,37]. Females have higher levels of endogenous glucocorticoids, and it would be interesting to further explore this aspect [38]. Given the small sample size in TCGA analysis, it may be worthwhile to explore age-related differences in differentially expressed genes and pathways with a larger cohort. Perhaps the addition of an age cutoff of 50 years to the staging system for ACC should be considered if further investigations have been completed in independent cohorts with similar findings.

This study is inherently limited by the retrospective nature of the data used in the analysis. The NCDB does not include variables such as cancer-specific survival, specifics of treatments administered, hormonal production by tumors, and molecular/genetic data. Numerous patients are also missing stage and grade information in this dataset, which limits the interpretability of multivariable models. Complete-case analysis may introduce selection bias if missing data are not random. However, sensitivity analyses yielded similar results, suggesting that missing data did not substantially affect the findings. It is important to note that age is strongly associated with non-cancer mortality, and without cancer-specific survival in this dataset, the observed effect may reflect competing risks rather than tumor-specific biology. Additionally, the Kaplan–Meier curve in Figure 5, which represents an unadjusted comparison, did not demonstrate a statistically significant difference between age cohorts; however, in multivariable Cox regression (Table 5), the association between outcome and age cohort was statistically significant, with this model accounting for imbalances in baseline prognostic factors between cohorts. After adjustment, the association between age cohort and outcome reached statistical significance, suggesting that confounding variables attenuated the crude association in the Kaplan–Meier analysis. Adjustment for these covariates also likely improved the precision of the estimated effect, contributing to the observed difference in statistical significance. Despite offering comprehensive genomic and transcriptomic data, TCGA has notable limitations, including a small sample size, lack of patient diversity and thus generalizability, and heterogeneity in tumor samples. Given the small sample size, a type II error is possible. However, the combined analysis provided insight into ACC genetics and gene expression profile by age. Despite these limitations, given the rarity of this malignancy, these are invaluable sources of data to further our understanding and guide future in vivo and in vitro investigations.

In summary, age ≥50 years is an independent factor associated with shorter OS in ACC. Although increasing age is associated with more comorbid conditions, other tumor-related factors not seen by the omics in this study, such as changes in immunogenic response to tumors or the tumor microenvironment, may contribute to the worse prognosis in older patients, resulting in lower treatment response or faster disease progression.

## 5. Conclusions

While age ≥50 years is an independent factor associated with shorter OS in ACC, no differences in clinical characteristics or multi-omic profiles were observed by age in the examination of two cohorts. Further research is necessary to explore how age plays a role in ACC to improve outcomes for these patients.

## Figures and Tables

**Table 1:** Baseline characteristics of patients with ACC reported in the NCDB between 2004 and 2022—full cohort.

	Overall
Total cases	2834
Age, median (range), years	56 (18–90)
Female patient (%)	1719 (60.7)
Race	
White (%)	2380 (84)
Black (%)	296 (10)
Asian and Pacific Islander (%)	91 (3)
Hispanic (%)	324 (11)
CDCI score	
0 (%)	2054 (72)
1 (%)	511 (18)
2 (%)	156 (6)
3 (%)	111 (4)
Stage at diagnosis	
I (%)	59 (2)
II (%)	231 (8)
III (%)	74 (3)
IV (%)	445 (16)
Unknown (%)	2025 (71)
Tumor grade	
1–2 (%)	110 (4)
3–4 (%)	260 (9)
Unknown	2464 (87)
Surgery (%)	1960 (69)
Systemic therapy (%)	1278 (45)
Radiation (%)	500 (18)
Mortality (%)	1713 (60)

**Table 2:** Comparative data between NCDB cohorts—men and pre- and post-menopausal women.

	Men (1115)	Pre-Menopausal (486)	Post-Menopausal (937)	*p*-Value ^(a,b)^
Age, Median (range), Years	56 (18–90)	35 (18–45)	67 (55–90)	<0.001, <0.001
Race				1.00, 1.00
White (%)	947 (85)	405 (83)	798 (85)	
Black (%)	93 (8)	52 (11)	105 (11)	
Asian and Pacific Islander (%)	47 (4)	9 (2)	23 (2)	
Hispanic (%)	130 (12)	77 (16)	73 (8)	0.02, 0.004
CDCI Score				<0.001, 0.23
0–1 (%)	998 (90)	469 (97)	823 (89)	
2–3 (%)	117 (10)	17 (3)	114 (11)	
Stage				0.51, 0.10
1–2 (%)	94 (8)	48 (10)	114 (12)	
3–4 (%)	198 (18)	87 (18)	178 (19)	
N/A ^c^	823 (74)	351 (72)	645 (69)	
Tumor Grade				0.072, 0.42
1–2 (%)	43 (4)	13 (3)	37 (4)	
3–4 (%)	115 (10)	42 (9)	77 (8)	
N/A	957 (86)	431 (88)	823 (88)	
Surgery (%)	750 (67)	370 (76)	620 (66)	<0.001, 0.64
R0 Resection (%)	508 (46)	267 (55)	417 (45)	1.00, 1.00
Systemic Therapy (%)	502 (45)	284 (58)	353 (38)	<0.001, <0.001
Radiation (%)	191 (17)	93 (19)	167 (18)	0.36, 0.68

^a.^ Comparison between men and pre-menopausal women *p*-value. ^b.^ Comparison between men and post-menopausal women *p*-value. ^c.^ N/A = not available.

**Table 3:** Cox proportional-multivariable hazards regression model for data from Table 2 (*n* = 2538).

Variable	Hazard Ratio (HR)	95% Confidence Interval for HR	*p*-Value
Men			**0.016**
Pre-menopausal women	0.268	0.105–0.689	**0.006**
Post-menopausal women	0.912	0.493–1.688	0.770
CDCI Score	0.768	0.321–1.841	0.554
Tumor Grade	1.793	0.968–3.320	0.063
Stage	1.064	0.604–1.874	0.829
Resection	1.799	0.968–3.344	0.063

Bold indicates a statistically significant *p*-value.

**Table 4:** Comparative data between NCDB age cohorts.

	Age < 50 (973)	Age ≥ 50 (1861)	*p*-Value
Age, mean (range), years	37 (18–49)	64 (50–90)	
Female (%)	595 (61)	1124 (60)	0.71
Race			**0.006**
White (%)	802 (82)	1578 (85)	
Black (%)	108 (11)	188 (10)	
Asian and Pacific Islander (%)	27 (3)	64 (3)	
Hispanic (%)	142 (15)	182 (10)	**<0.001**
CDCI Score			**<0.001**
0–1 (%)	927 (95)	1640 (88)	
2–3 (%)	46 (5)	221 (12)	
Stage			0.69
1–2 (%)	87 (9)	203 (11)	
3–4 (%)	164 (17)	355 (19)	
N/A ^a^	722 (74)	1303 (70)	
Tumor Grade			0.91
1–2 (%)	37 (4)	73 (4)	
3–4 (%)	90 (9)	170 (9)	
N/A	846 (87)	1618 (87)	
Surgery (%)	720 (74)	1240 (67)	**<0.001**
R0 Resection (%)	509 (52)	839 (45)	0.90
Systemic Therapy (%)	537 (55)	741 (40)	**<0.001**
Radiation (%)	170 (17)	330 (18)	0.88

a. N/A = Not available. Bold indicates significant *p*-values.

**Table 5:** Cox proportional-multivariable hazards regression model for data from Table 4 comparing patients <50 years (973) to patients ≥50 years (1861).

Variable	Hazard Ratio	95% Confidence Interval	*p*-Value
Age	1.559	1.403–1.733	**<0.001**
Hispanic	1.062	0.917–1.230	0.424
CDCI Score	1.197	1.019–1.405	**0.028**
Surgery	0.231	0.209–0.256	**<0.001**
Systemic Treatment	0.977	0.885–1.078	0.645

Bold indicates significant *p*-values.

**Table 6:** Comparative data between TCGA age cohorts.

	Age < 50 (47)	Age ≥ 50 (45)	*p*-Value
Recurrence (%)	23 (51)	21 (55)	0.83
Mortality (%)	14 (30)	20 (44)	0.20
C1A (%)	20 (51)	23 (59)	0.65
C1B (%)	19 (49)	16 (41)	
Intermediate-high methylation (%)	22 (55)	25 (64)	0.49
Stage			0.52
1–2 (%)	29 (63)	24 (55)	
3–4 (%)	17 (37)	20 (45)	
Metastasis present (%)	8 (17)	10 (23)	0.60
Excess adrenal hormone (%)	32 (73)	21 (54)	0.11
Cortisol ^1^	23	15	0.18
Other hormones	9	7	
Grade			1.00
1–2 (%)	9 (21)	6 (21)	
3–4 (%)	34 (79)	22 (79)	
Mitotane (%)	29 (66)	26 (60)	0.66

^1^ Includes cortisol alone and with other hormones.

## Data Availability

Some of the data used in this study were obtained from the National Cancer Database (NCDB), which is not publicly available. Access requires approval from the American College of Surgeons and is limited to eligible researchers at CoC-accredited institutions. The other data in this study are publicly available from The Cancer Genome Atlas (TCGA) Adrenocortical Carcinoma (ACC) Firehose Legacy cohort. Processed data were accessed through the cBioPortal for Cancer Genomics (https://www.cbioportal.org) and originally generated by the TCGA Research Network (https://www.cancer.gov/. accessed on 17 April 2025).

## Supplementary Materials

The following supporting information can be downloaded at https://www.mdpi.com/article/10.3390/cancers18091483/s1. Supplemental Figure S1: Kaplan–Meier curve depicting OS of men and women stratified by age and menopausal status; Supplemental Table S1: TCGA driver mutation by age cohort; Supplemental Figure S2: Differentially expressed pathways in the TCGA ACC dataset by age cohort; Supplemental Table S2: Known Stage Sensitivity Analysis Referencing Table 3; Supplemental Table S3: Known CDCI Score Sensitivity Analysis Referencing Table 3; Supplemental Table S4: Known CDCI Score Sensitivity Analysis Referencing Table 5; Supplemental Table S5: Known Stage Sensitivity Analysis Referencing Table 5.

## Abbreviations

The following abbreviations are used in this manuscript:

ACC	Adrenocortical carcinoma
AJCC	American Join Committee on Cancer
CDCI	Charleson–Deyo Comorbidity Index
DTC	Differentiated thyroid cancer
GSEA	Gene set enrichment analysis
NCDB	National Cancer Database
NIDAP	NIH Integrated Analysis Platform
OS	Overall survival
ROC	Receiver operating characteristic
TCGA	The Cancer Genome Atlas
