# Supplementary material for: Association of Age with Outcomes in Adrenocortical Carcinoma: A Combined Cancer Registry and Multi-Omic Analysis
Source: Cancers (Basel). 2026 May 5;18(9):1483. doi: 10.3390/cancers18091483 (PMC13162810; doi:10.3390/cancers18091483)

HALLMARK_BILE_ACID_METABOLISM

H: hallmark gene sets, Under50−Over50, ES=0.34, NES=1.55, pval=0.0055, padj=0.046

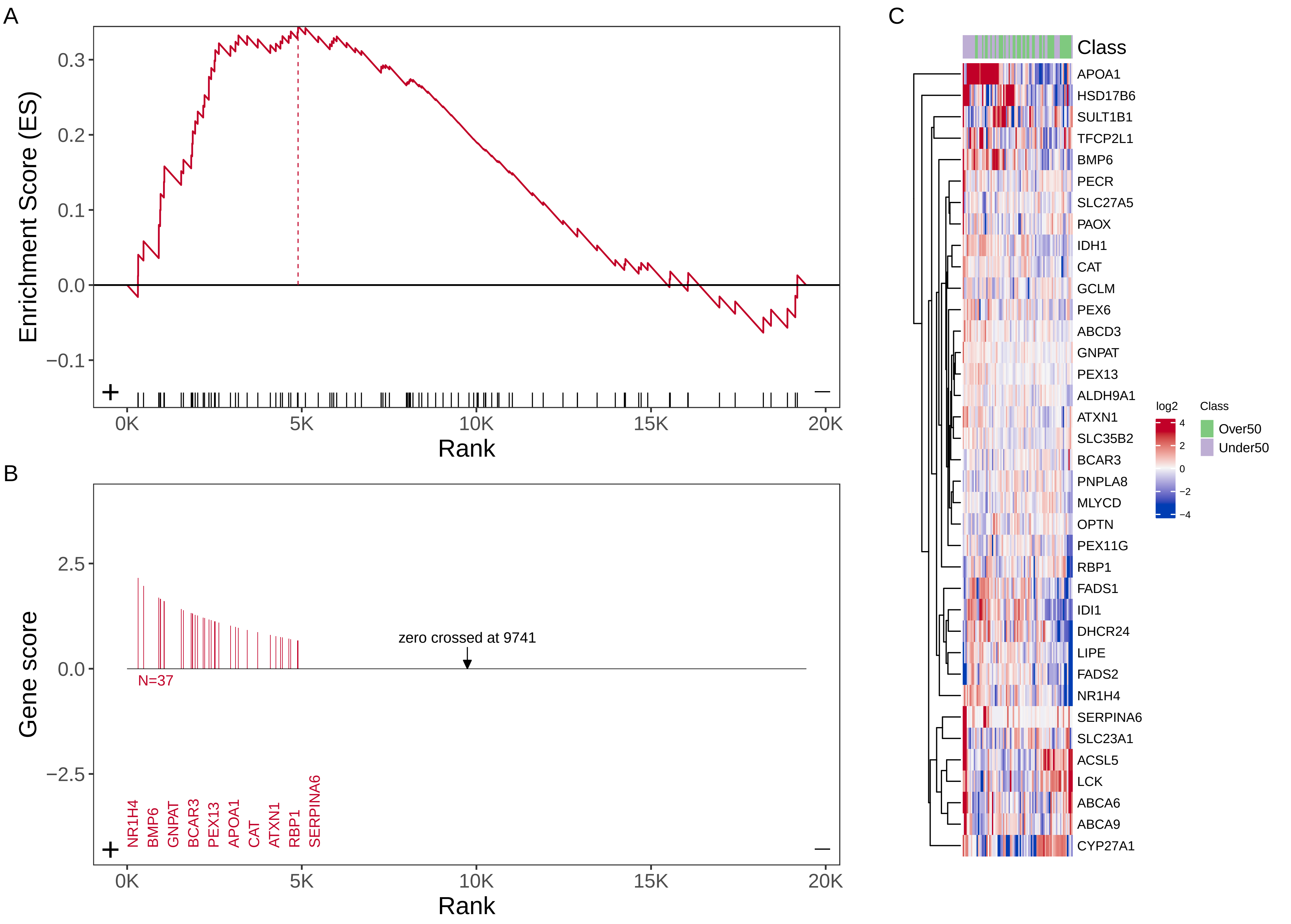

HALLMARK_CHOLESTEROL_HOMEOSTASIS

H: hallmark gene sets, Under50−Over50, ES=0.44, NES=1.86, pval=0.0012, padj=0.02

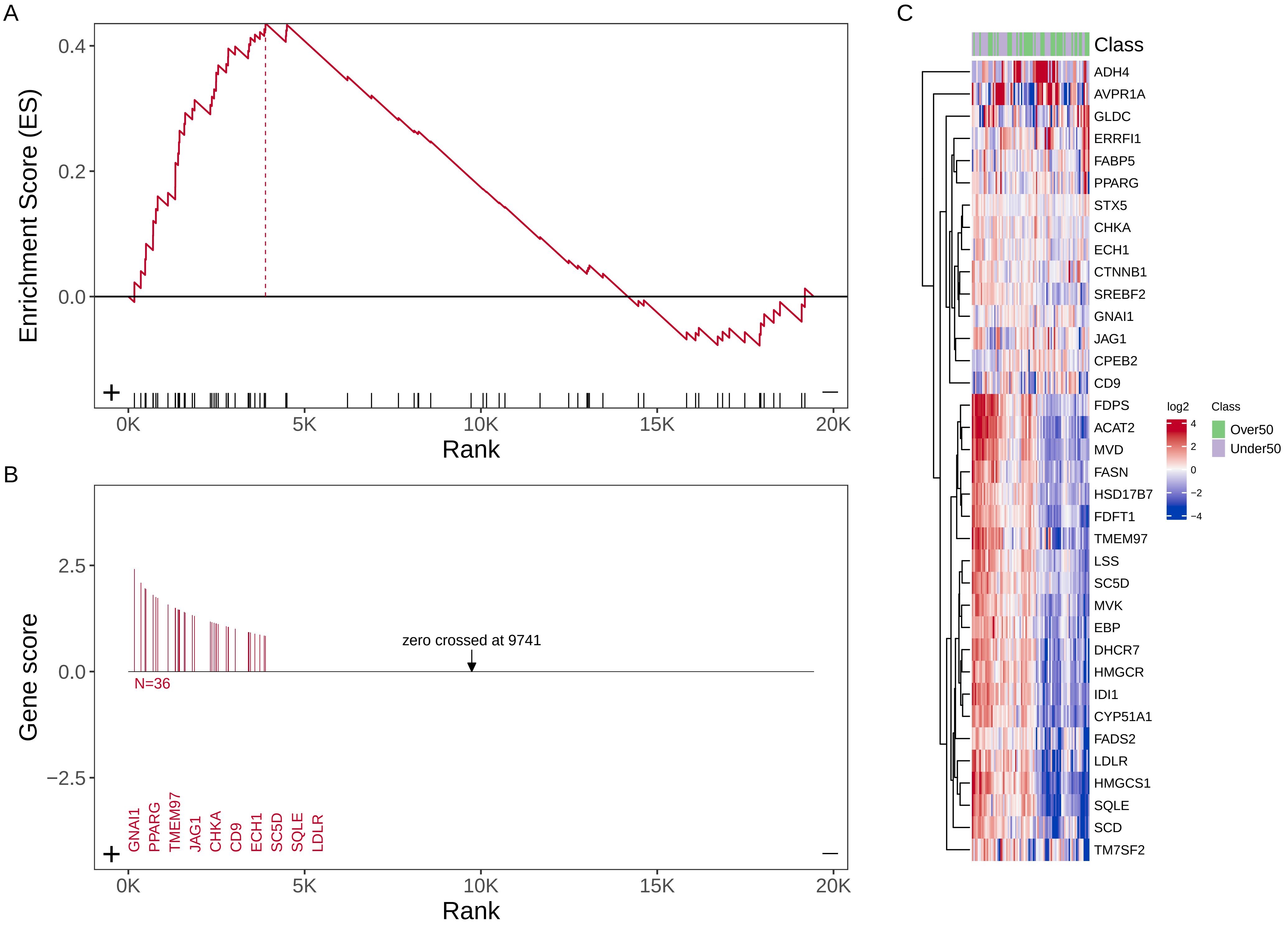

HALLMARK_FATTY_ACID_METABOLISM

H: hallmark gene sets, Under50−Over50, ES=0.32, NES=1.55, pval=0.0024, padj=0.03

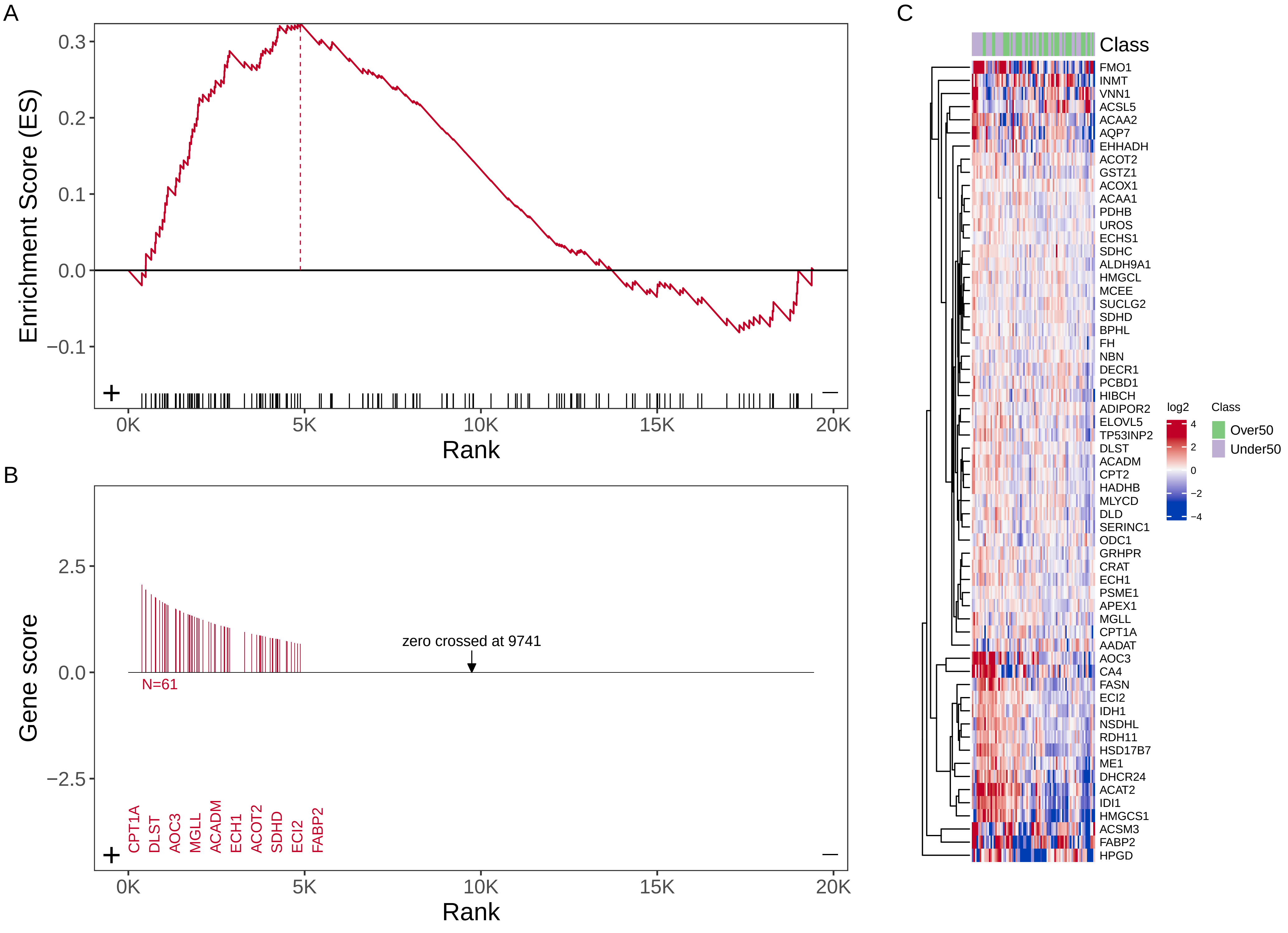

HALLMARK_HYPOXIA

H: hallmark gene sets, Under50−Over50, ES=−0.38, NES=−1.88, pval=0.0004, padj=0.01

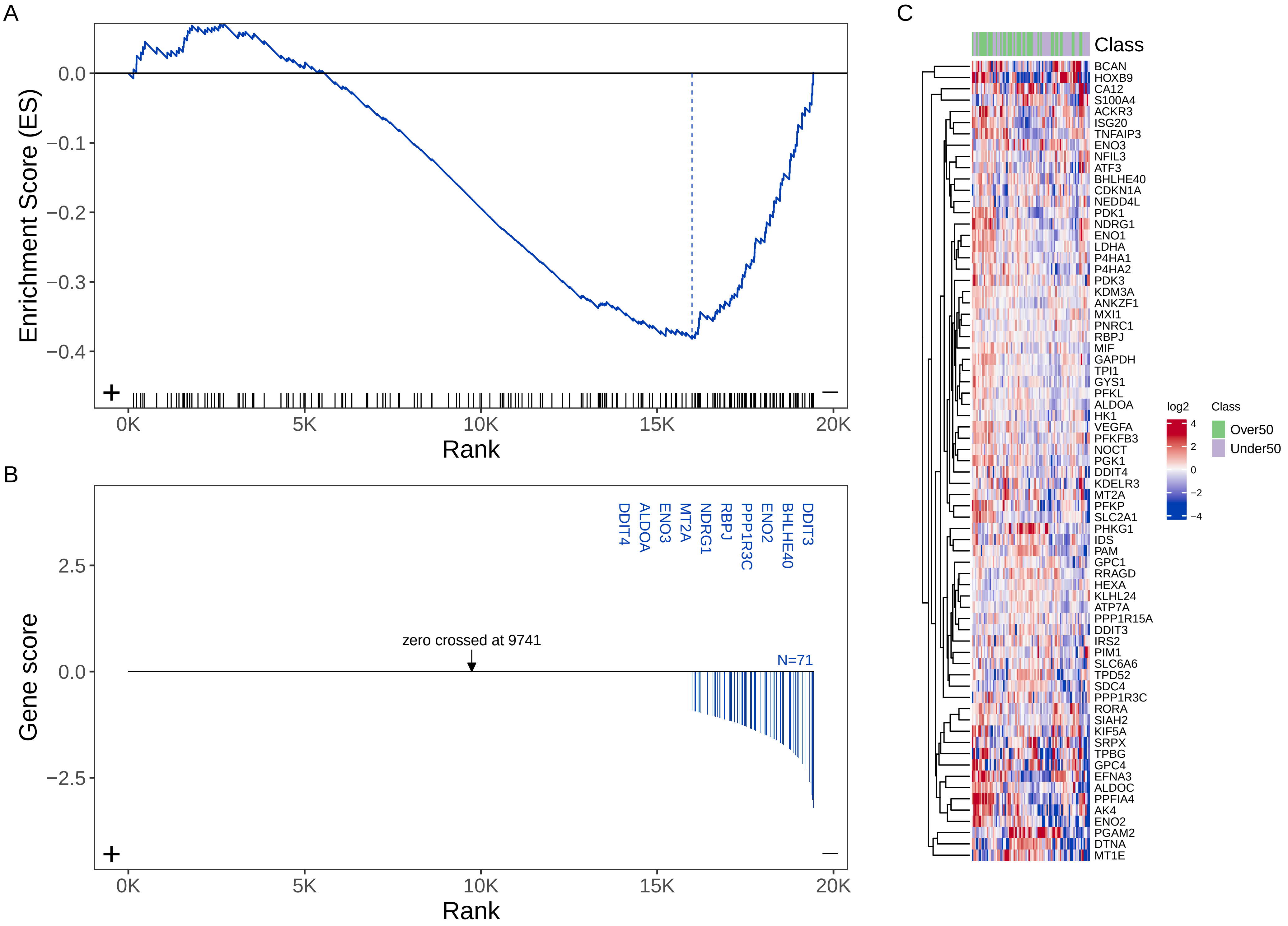

HALLMARK_P53_PATHWAY

H: hallmark gene sets, Under50−Over50, ES=−0.38, NES=−1.86, pval=0.0004, padj=0.01

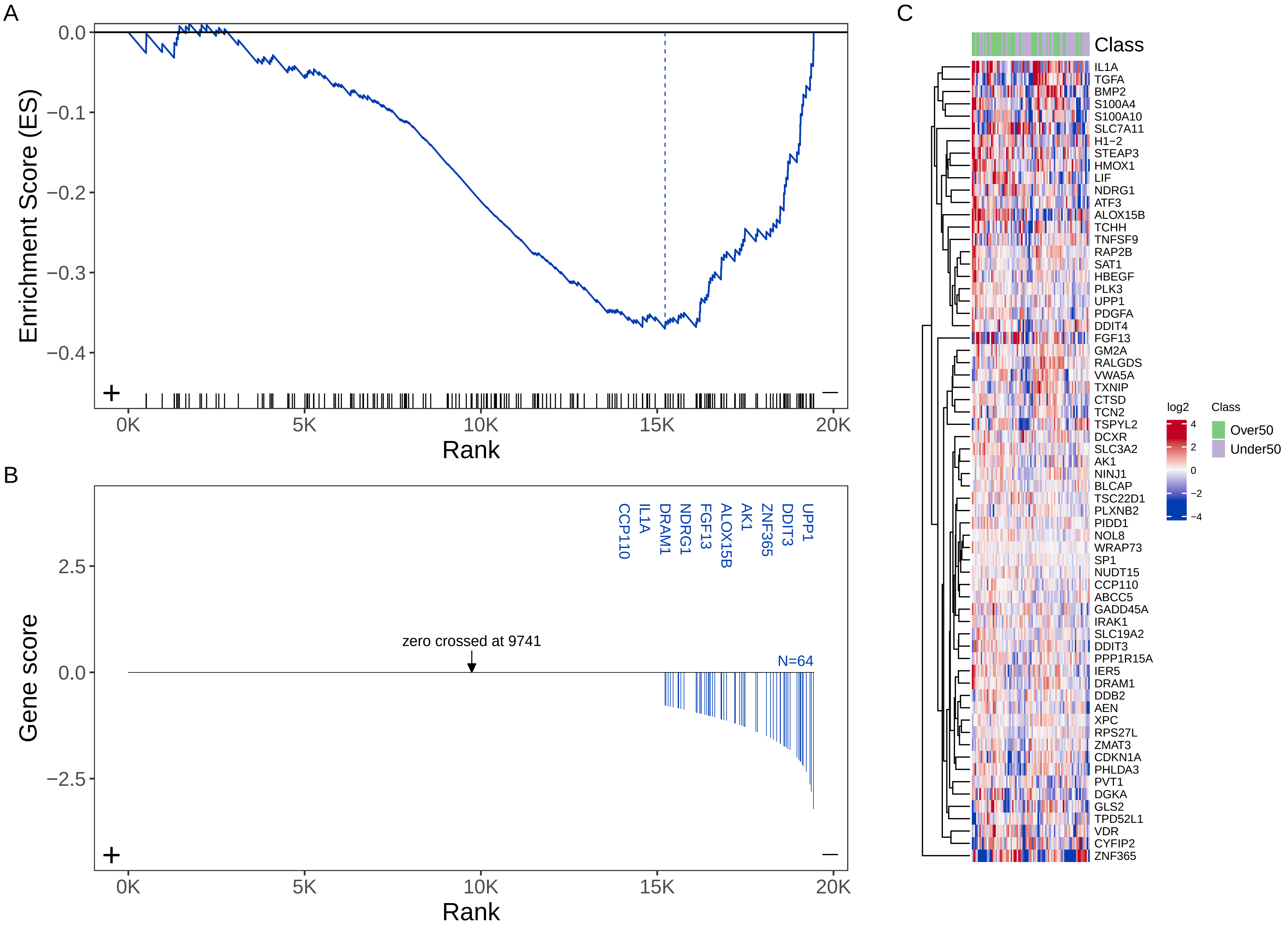

HALLMARK_PEROXISOME

H: hallmark gene sets, Under50−Over50, ES=0.35, NES=1.55, pval=0.0055, padj=0.046

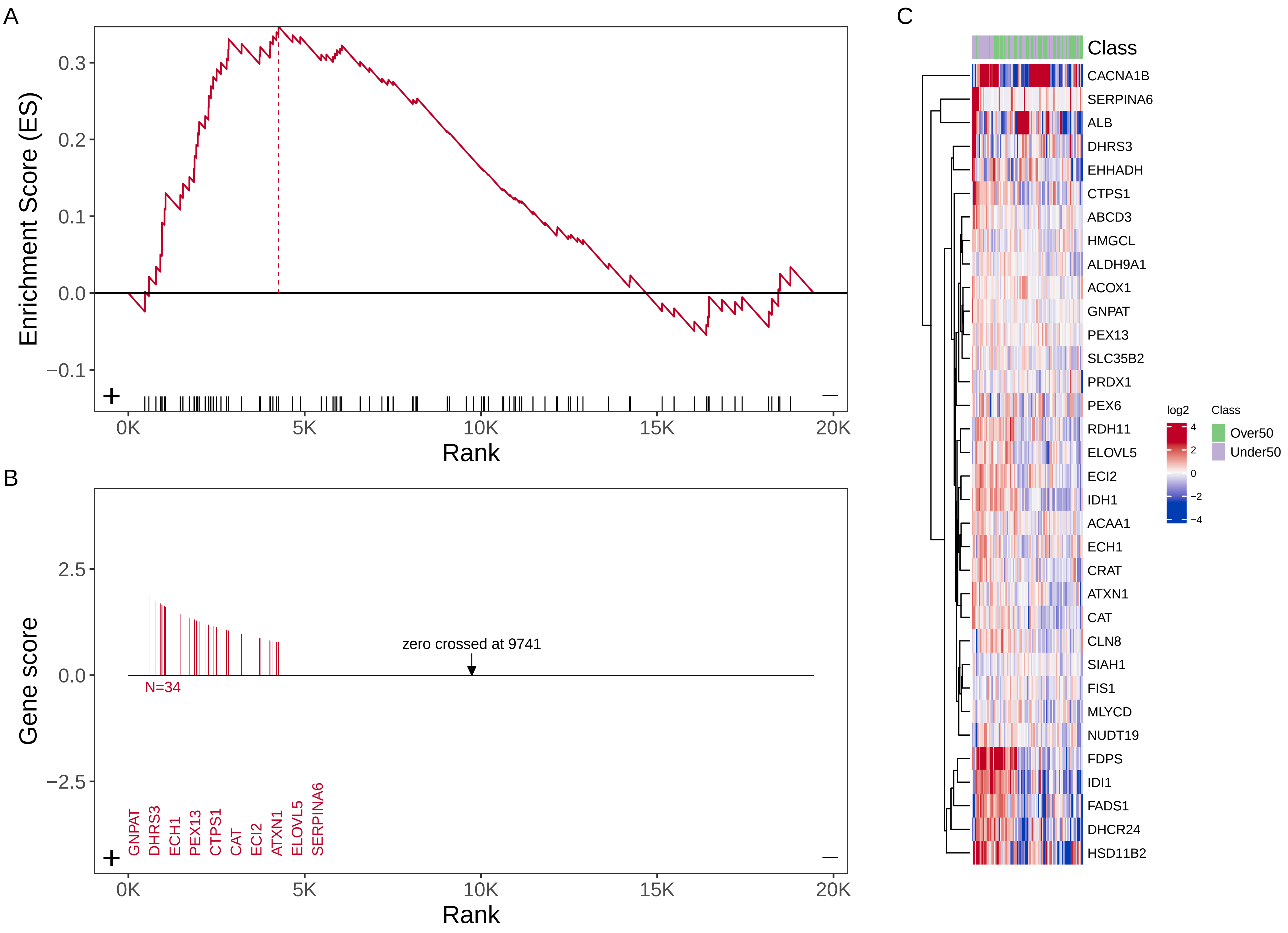

Supplement: Supplementary file 1 [file cancers-18-01483-s001.zip › Supplemental Figure S2 Differentially Expressed Pathways.pdf]
