# Supplementary figures and images for: Association of Age with Outcomes in Adrenocortical Carcinoma: A Combined Cancer Registry and Multi-Omic Analysis
Source: Cancers (Basel). 2026 May 5;18(9):1483. doi: 10.3390/cancers18091483 (PMC13162810; doi:10.3390/cancers18091483)

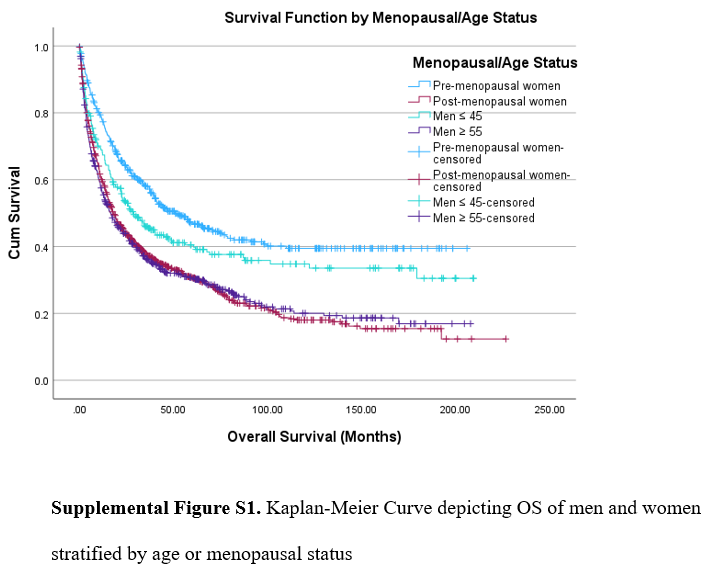

Supplement: Supplementary file 1 [file cancers-18-01483-s001.zip › Supplemental Figure S1 KM Curve Men and Women split into menopausal age groups.png]

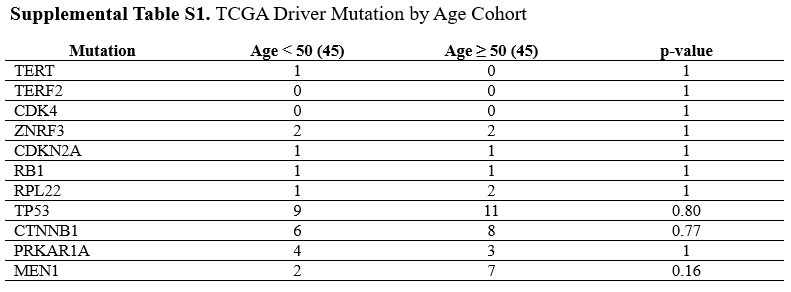

Supplement: Supplementary file 1 [file cancers-18-01483-s001.zip › Supplemental Table S1 TCGA Driver Mutation by Age Cohort.png]

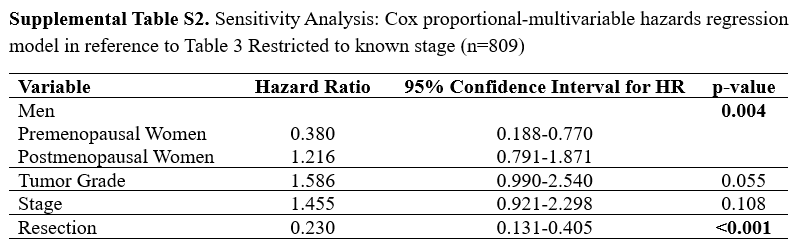

Supplement: Supplementary file 1 [file cancers-18-01483-s001.zip › Supplemental Table S2 Stage Sensitiviy Analysis Referencing Table 3.png]

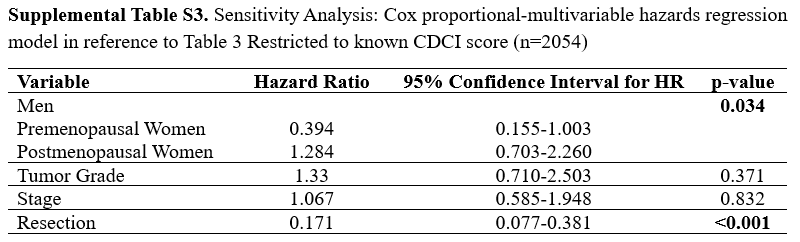

Supplement: Supplementary file 1 [file cancers-18-01483-s001.zip › Supplemental Table S3 CDCI Sensitivity Analysis Referencing Table 3.png]

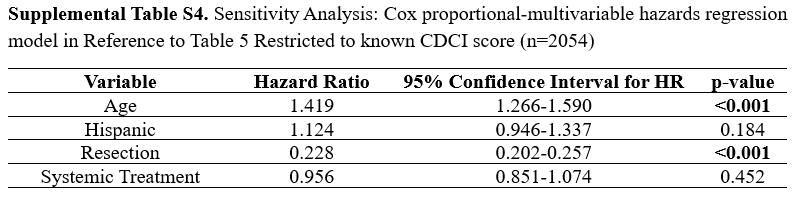

Supplement: Supplementary file 1 [file cancers-18-01483-s001.zip › Supplemental Table S4 CDCI Sensitivity Analysis Referencing Table 5.png]

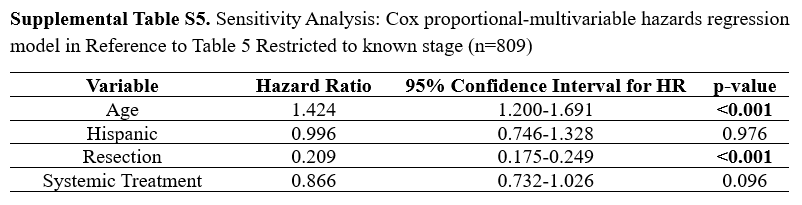

Supplement: Supplementary file 1 [file cancers-18-01483-s001.zip › Supplemental Table S5 Stage Sensitivity Analysis Referencing Table 5.png]
